# CoSinGAN: Learning COVID-19 Infection Segmentation from a Single Radiological Image

**DOI:** 10.3390/diagnostics10110901

**Published:** 2020-11-03

**Authors:** Pengyi Zhang, Yunxin Zhong, Yulin Deng, Xiaoying Tang, Xiaoqiong Li

**Affiliations:** 1 School of Life Science, Beijing Institute of Technology, Haidian District, Beijing 100081, China; zhangpybit@gmail.com (P.Z.); bityunxinz@gmail.com (Y.Z.); deng@bit.edu.cn (Y.D.); xiaoying@bit.edu.cn (X.T.); 2 Key Laboratory of Convergence Medical Engineering System and Healthcare Technology, Ministry of Industry and Information Technology, Haidian District, Beijing 100081, China

**Keywords:** covid19 infection segmentation, generative model, single radiological image, conditional distribution

## Abstract

Computed tomography (CT) images are currently being adopted as the visual evidence for COVID-19 diagnosis in clinical practice. Automated detection of COVID-19 infection from CT images based on deep models is important for faster examination. Unfortunately, collecting large-scale training data systematically in the early stage is difficult. To address this problem, we explore the feasibility of learning deep models for lung and COVID-19 infection segmentation from a single radiological image by resorting to synthesizing diverse radiological images. Specifically, we propose a novel conditional generative model, called CoSinGAN, which can be learned from a single radiological image with a given condition, i.e., the annotation mask of the lungs and infected regions. Our CoSinGAN is able to capture the conditional distribution of the single radiological image, and further synthesize high-resolution (512 × 512) and diverse radiological images that match the input conditions precisely. We evaluate the efficacy of CoSinGAN in learning lung and infection segmentation from very few radiological images by performing 5-fold cross validation on COVID-19-CT-Seg dataset (20 CT cases) and an independent testing on the MosMed dataset (50 CT cases). Both 2D U-Net and 3D U-Net, learned from four CT slices by using our CoSinGAN, have achieved notable infection segmentation performance, surpassing the COVID-19-CT-Seg-Benchmark, i.e., the counterparts trained on an average of 704 CT slices, by a large margin. Such results strongly confirm that our method has the potential to learn COVID-19 infection segmentation from few radiological images in the early stage of COVID-19 pandemic.

## 1. Introduction

The highly contagious Coronavirus Disease 2019 (COVID-19), caused by the severe acute respiratory syndrome coronavirus 2 (SARS-CoV-2) virus [1,2,3], has spread rapidly across the world and millions of people have been infected. This surge in infected patients has overwhelmed healthcare systems in a short time. Due to the close contact with patients, many medical professionals have also been infected, further worsening the healthcare situation. Globally, as of 3:24 p.m. CEST, 8 October 2020, there have been 36,002,827 confirmed cases of COVID-19, including 1,049,810 deaths, reported to World Health Organization (WHO) [4]. Early detection of COVID-19 is significantly important to prevent the spread of this epidemic.

Reverse transcription polymerase chain reaction (RT-PCR) is the de facto golden standard for COVID-19 diagnosis [5,6]. However, the global shortage in RT-PCR test kits has severely limited the extensive detection of COVID-19. Meanwhile, the current clinical experience implies RT-PCR has a low sensitivity [7,8,9], especially in the early outbreak of COVID-19. This means that multiple testing may be required to rule out the false negative cases [10], which may delay the confirmation of suspected patients and take up huge healthcare resources.

Since most patients infected by COVID-19 are initially diagnosed with pneumonia [11], radiological examinations, including computed tomography (CT) and X-rays, are able to provide visual evidence of COVID-19 infection for diagnosis and patient triage. Existing chest CT findings in COVID-19 infection [12] have implied that chest CT screening on patients in the early stage of COVID-19 presents superior sensitivity over RT-PCR [8] and even confirms the false negative cases given by RT-PCR [5]. Therefore, radiological examinations are currently used as parallel testing in COVID-19 diagnosis. However, as the number of infected patients dramatically increases, clinicians need to analyze radiographs repeatedly, which brings huge pressure to them. Therefore, there is an immediate need for developing methods for automated infection measurement and COVID-19 diagnosis based on radiological images to reduce the efforts of clinicians and accelerate the diagnosis process.

Many approaches, mostly using deep models, have been proposed for automated COVID-19 diagnosis based on chest CT [10,13,14,15] or chest X-ray [11,16], and have claimed notable detection accuracy of COVID-19 infection. However, the research of these approaches tends to lag slightly behind the outbreak of COVID-19 pandemic. It is probably because accumulating sufficient radiological images that are required to train deep models is difficult in the early stage of COVID-19 pandemic. To solve the dilemma of training deep models on insufficient training samples and realize automated COVID-19 diagnosis in the early stage, some research resorts to a shallow network [11], prior knowledge [11], transfer learning [16,17], and data augmentation method based on a generative adversarial network (GAN) [17,18]. However, these methods still require a relatively large training dataset, and thus cannot respond immediately to the outbreak of the COVID-19 pandemic.

The COVID-19-CT-Seg-Benchmark [19], to our best knowledge, is the first attempt to explore annotation-efficient methods for COVID-19 CT scan segmentation by focusing on left lung, right lung and infection segmentation from three perspectives: (1) learning with limited annotations; (2) learning to segment COVID-19 CT scans from non-COVID-19 CT scans; and (3) learning with both COVID-19 and non-COVID-19 CT scans. According to the benchmark results [19] (https://gitee.com/junma11/COVID-19-CT-Seg-Benchmark), the gains in COVID-19 infection segmentation performance from learning with other lung lesion CT scans (e.g., lung cancer and pleural effusion) are rather limited, or, to be more precise, are negative. Thus, we mainly focus on the task of learning with limited annotations. To be more annotation-efficient, we further make the task more challenging: learning with limited CT slices instead of limited CT volumes. In particular, we aim to explore the feasibility of learning automated lung and COVID-19 infection segmentation from a single radiological image by resorting to generating diverse labeled radiological images.

GAN is one of the most famous generative models, and is able to generate realistic images that are indistinguishable from real ones in limited domains through adversarial learning [20]. GAN consists of two components, i.e., a generator and a discriminator. The generator is trained by minimizing the image reconstruction loss and adversarial loss simultaneously, thereby fooling the discriminator to maximize the probability of generated image being classified as real images. GAN has been widely used in various computer vision tasks, such as image synthesis [21,22], image resolution [23], image restoration [24], image editing [25], and image-to-image translation [26]. However, training these GAN models [21,22,23,24,25,26] requires tens of thousands of images [27]. Several recent works have proposed to ‘overfit’ a deep model to a single training example [27,28,29,30]. Specifically, Zhou et al. [28] proposed a generative model to be trained on a single texture image, and used it for example-based non-stationary texture synthesis. Unlike [28], Shocher et al. [29] introduced an internal GAN (InGAN) to be trained on a single natural image, and illustrated it in the context of retargeting. To expand the applications of single image generative model, Shaham et al. [30] designed a pure generative model, i.e., SinGAN, by mapping noise to image, and illustrated it in a wide range of image manipulation tasks such as super-resolution, paint-to-image, harmonization, and single image animation. Moreover, to make the training process more tractable, Hinz et al. [27] proposed several improved techniques for training single-image GANs. However, these methods are not suitable for our task because they cannot be learned easily from a single radiological image to get the labeled radiological images by synthesizing the lungs and infected regions at any given positions. In other words, we need to learn a generative model that is conditioned on the positions of the lungs and infected regions from a single radiological image.

**Figure 1:**
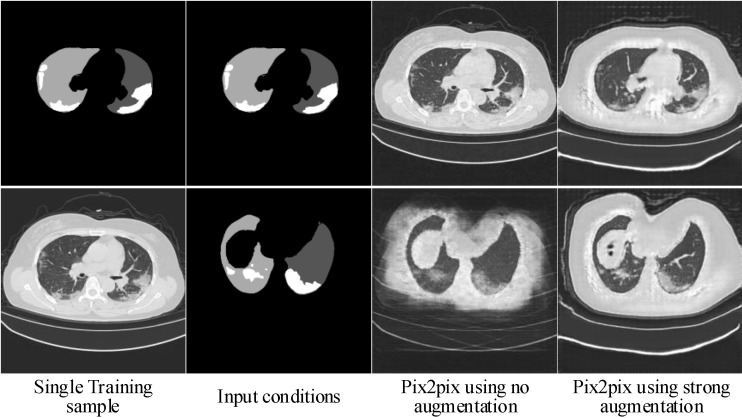
Synthetic radiological images from two pix2pix models trained separately by using strong and weak (or no) augmentation. The white and gray (e.g., light gray and dark gray) in condition masks represent the infected regions and lungs, respectively.

Learning such a conditional generative model from a single image is very challenging. One should prevent the generator from being ‘overfitted’ to the single input condition, and, meanwhile, need to ‘overfit’ the generator to the single training image as much as possible. The former aims to make the generator can generalize to different conditions, whereas the latter aims at reconstructing clear and accurate image details. One of the key points for learning such a conditional generative model is image augmentation, which is not so critical for SinGAN [30] or for the GANs [21,22,23,24,25,26] trained on large datasets. Strong data augmentation is an effective approach to avoid overfitting, whereas it may corrupt the real data distribution and put an additional learning burden on the generator, thus leading to blurry image details. In comparison, weak (or no) data augmentation helps to reduce the fitting error, whereas it may make the generator not be able to generalize to different input conditions. Pix2pix [26] is a well-known image-to-image translation framework. To illustrate the conflict between fitting images and fitting conditions, we adapt pix2pix to our task by mapping the annotation mask of the lungs and infected regions to radiological image, and train two pix2pix models by using weak augmentation and strong augmentation, respectively. As illustrated in Figure 1, by using weak augmentation, the pix2pix model can produce high-quality images when the input conditions are close to the training one, whereas, when the input conditions are different from the training one, it fails to reconstruct image details accurately. In contract, by using strong augmentation, the pix2pix model can produce synthetic images with similar quality in spite of being blurry. To sum up, weak augmentation helps to fit images, whereas strong augmentation helps to fit conditions. Handling the balance between fitting image and fitting condition is very challenging, and, more importantly, the images produced by the pix2pix model look far from perfect.

To address these challenges, we propose a novel conditional generative model, called CoSinGAN, which can be learned from a single radiological image with a condition, i.e., the annotations of the lung and COVID-19 infection. Inspired by SinGAN [30], we build CoSinGAN with a pyramid of GANs, each of which is responsible for translating condition masks into radiological images on a different scale. We design a two-stage generator at each scale to cooperate with the multi-scale architecture by progressively adding image details and enhancing the condition constraints. A mixed reconstruction loss and a hierarchical data augmentation module are proposed to make the training of a pyramid of GANs more tractable. The former provides rich and stable gradient information for optimizing generators, while the latter produces data augmentation with different intensities for handling the balance between fitting condition and fitting image. Moreover, we provide an effective approach for CoSinGAN to generate diverse radiological images. Extensive ablation experiments strongly confirm the efficacy of our proposed methods. We evaluate the efficacy of CoSinGAN in learning lung and infection segmentation from few radiological images by performing 5-fold cross validation on a COVID-19-CT-Seg dataset (20 CT cases) and an independent test on MosMed dataset (50 CT cases). Both 2D U-Net and 3D U-Net [31,32] trained on synthetic images have achieved notable segmentation performance that are superior to the COVID-19-CT-Seg-Benchmark results. Note that our method uses only four real labeled slices, which are extremely less than an average of 704 real labeled images used in COVID-19-CT-Seg-Benchmark. Such results strongly confirm that our method has the potential to learn COVID-19 diagnosis from very few radiological images in the early stage of the COVID-19 pandemic. Our codes will be made publicly available at https://github.com/PengyiZhang/CoSinGAN.

## 2. Material and Methodology

### 2.1. Methodology

CoSinGAN consists of three key components, including multi-scale architecture with a pyramid of two-stage GANs, a mixed reconstruction loss, and a hierarchical data augmentation module.

#### 2.1.1. Multi-Scale Architecture with a Pyramid of Two-Stage GANs

**Figure 2:**
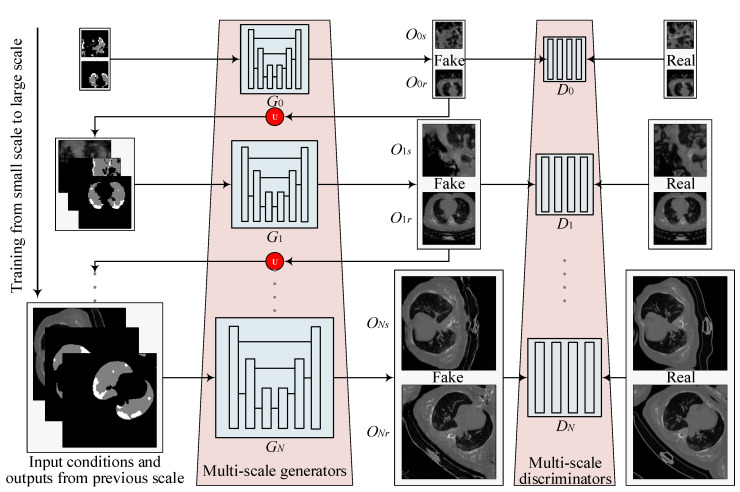
Multi-scale architecture of proposed CoSinGAN. CoSinGAN consists of a pyramid of GANs, each of which has a two-stage generator and is responsible for translating condition mask into radiological image at a different scale. The input to $G_{i}$ is an augmented condition mask and the synthetic radiological image from the scale i−1 that is upsampled to the scale *i* (except for scale 0). By iterating optimizations from small image scale to large image scale, CoSinGAN progressively learns to generate realistic and high-resolution (512 × 512) radiological images that match the input condition masks precisely. The notation U represents an upsampling operation. The white and gray in the condition masks represent the infected regions and lungs, respectively.

**Figure 3:**
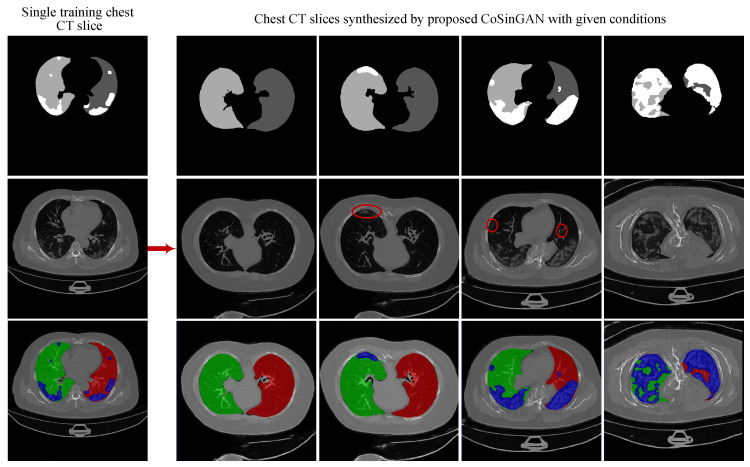
High-resolution (512 × 512) synthetic radiological images from proposed CoSinGAN. CoSinGAN is a novel conditional generative model trained on a single chest CT slice. Our model is able to capture the conditional distribution of visual finds of the lungs and COVID-19 infection accurately. We explore the feasibility of learning deep models for automated lung and COVID-19 infection segmentation from a single radiological image by resorting to synthesizing diverse radiological images. Both 2D U-Net and 3D U-Net trained on synthetic images achieves notable segmentation performance. The first row denotes the input conditions, where the white and gray (e.g., light gray and dark gray) represent the infected regions and lungs, respectively. The last row denotes synthetic CT slices with input condition overlays, where the blue denotes infected regions and the red (or green) represents the lungs.

**Figure 4:**
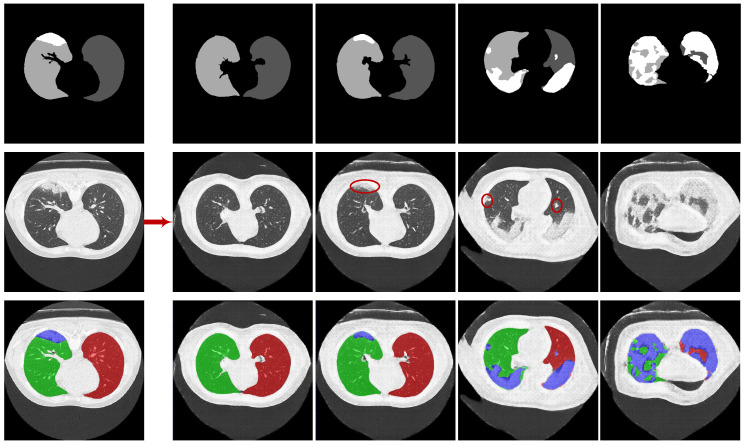
The synthetic radiological images from CoSinGAN trained with given axial CT slice and annotation mask of the lungs and COVID-19 infection. The first row denotes the conditional masks, where the white and gray (e.g., light gray and dark gray) represent the infected regions and lungs, respectively. The last row denotes synthetic CT slices with input condition overlays, where the blue denotes infected regions and the red (or green) represents the lungs.

**Overall.** Learning a GAN-based generative model to synthesize high-resolution images is very challenging due to the unstable adversarial training process. A useful trick is to learn a pyramid of GANs as adopted by SinGAN [30] to increase the image resolution progressively. We borrow this trick and build CoSinGAN with a multi-scale architecture as depicted in Figure 2. Note that we intend to use the synthetic radiological images to train deep models for automated lung and infection segmentation. Therefore, the synthetic radiological images should match the given input conditions precisely. To achieve that, CoSinGAN is designed to capture the ‘conditional’ distribution of a single radiological image rather than the ‘unconditional’ distribution of a single nature image as done by SinGAN. Learning conditional distribution from a single image is more challenging because one should pay more attention to preventing deep models from being ‘overfitted’ to the single input condition, and, meanwhile, need to ‘overfit’ them to the single training image as much as possible. To tackle this problem, at each scale, we design a two-stage GAN to cooperate with the pyramid hierarchy. The first stage is mainly responsible for fitting the input condition and increasing the image resolution, while the second stage is responsible for restoring image details that may not be reconstructed accurately in the first stage. Through iterative optimization between enhancing condition constraints and restoring image details across all image scales, our CoSinGAN can generate realistic and high-resolution radiological images that match the given input conditions precisely as illustrated in Figure 3 and Figure 4.

**Multi-scale architecture.** As shown in Figure 2, CoSinGAN consists of N+1 GANs, i.e., multi-scale generators $\left\{G_{0},G_{1},\ldots ,G_{N}\right\}$ and multi-scale discriminators $\left\{D_{0},D_{1},\ldots ,D_{N}\right\}$, corresponding to N+1 different image scales. The original single training image $X_{orig}$ and its condition mask $C_{orig}$ are initially resized to the pre-defined image scales respectively to conduct the training sample $\left\{X_{i},C_{i}|i\in \left[0,N\right]\right\}$ for N+1 GANs. The training of CoSinGAN starts from the coarsest image scale 0, and gradually passes through all image scales. Note that we only train one stage of a GAN at a time and the other GANs are fixed. For the generation of radiological images at the specific scale *i*, the generators $\left\{G_{0},G_{1},\ldots ,G_{N}\right\}$ are sequentially involved, where the output $O_{j-1}$ of $G_{j-1}$ is upsampled to the *j*-th image scale, and is further combined with $C_{j}$ to build the input of $G_{j}$ (j∈[1,i]). Benefiting from the output $O_{j-1}$ of previous generator $G_{j-1}$, $G_{j}$ will not fail quickly in the adversarial learning of GAN, and will continue to fight with $D_{j}$ and learn to generate realistic radiological image gradually. As there is no previous scale, $G_{0}$ learns to map the conditional mask $C_{0}$ into radiological image directly. Due to the small image scale, $G_{0}$ can be trained easily and further start the training of subsequent image scales smoothly. The multi-scale conditions $\left\{C_{i}|i\in \left[1,N\right])\right\}$, modulating the input of GANs across *N* image scales, will enforce the output of CoSinGAN to match the given conditions strictly.

**Figure 5:**
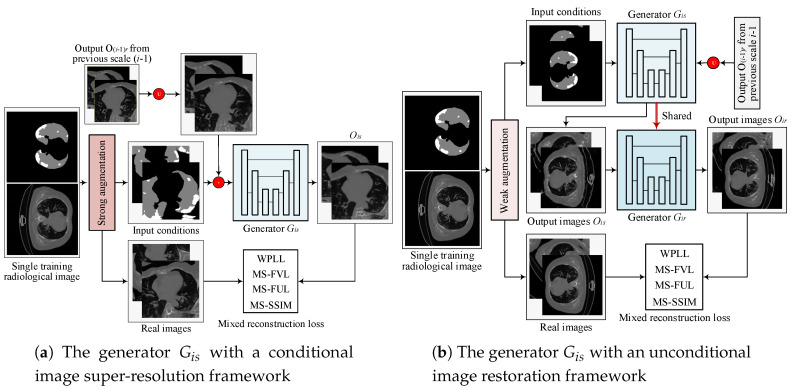
Illustration of the architecture of proposed two-stage GAN at the i-th image scale. The notation U represents an upsampling operation. The white and gray in the condition masks represent the infected regions and lungs, respectively.

**Two-stage GAN.** At a specific image scale *i*, we design a two-stage GAN as depicted in Figure 5. The generator $G_{i}$ in its first stage, called $G_{is}$, is designed to perform conditional image super-resolution, responsible for fitting the condition and increasing image resolution simultaneously. $G_{is}$ inputs the output $O_{i-1}$ from previous scale i−1 that is first upsampled to the *i*-th image scale and sequentially modulated by the condition $C_{i}$, and outputs an image $O_{is}$ with the *i*-th image scale:

(1) $$O_{is}=G_{is}\left(U_{i}\left(O_{i-1}\right)+C_{i}\right)$$

where $U_{i}$ denotes the upsampling operation. In the second stage, we directly copy the trained generator $G_{is}$ to perform unconditional image restoration, thus called $G_{ir}$. $G_{ir}$ inputs $O_{is}$ and outputs an image $O_{ir}$ with the *i*-th image scale:

(2) $$O_{ir}=G_{ir}\left(O_{is}\right)$$

Thus, the full image generation process of proposed two-stage GAN can be formulated as:

(3) $$O_{i}=G_{ir}\left(G_{is}\left(U_{i}\left(O_{i-1}\right)+C_{i}\right)\right)$$

We specially design a hierarchical data augmentation module, which can produce strong augmentation and weak augmentation (detailed in Section 2.1.3) to train such a two-stage GAN. We first perform strong augmentation on training sample $\left(X_{i},C_{i}\right)$ to train $G_{is}$ to make $G_{is}$ can generalize to different input conditions. Next, we do weak augmentation on training sample $\left(X_{i},C_{i}\right)$ to train $G_{ir}$ to make $G_{ir}$ restore image details as much as possible. The two-stage GAN is trained by a two-step optimization: (a) enhance given condition constraints but may blur image details, and (b) restore more image details but may violate given conditions. By iterating such a two-step optimization through all image scales progressively, CoSinGAN learns to generate high-resolution radiological images that match the given conditions well and have clear and accurate image details.

**Implementation details.** A total of nine image scales are used in our implementation of CoSinGAN for synthesizing high-resolution chest CT slices, including 32 × 32, 48 × 48, 64 × 64, 96 × 96, 128 × 128, 192 × 192, 256 × 256, 384 × 384, and 512 × 512. We purposely choose such image scales to facilitate the design of the multi-scale generators with different numbers of down-sampling layers. Specifically, we choose a network architecture similar with the pix2pix model [26], including a U-Net-style generator and a patch discriminator. Considering the reusability of trained models between two adjacent image scales, we set the number of 2 × downsampling layers in the U-Net-style generators of CoSinGAN to (4, 4, 5, 5, 6, 6, 7, 7, 7), respectively. Meanwhile, the numbers of convolutional layers in the corresponding discriminators are set to (6, 6, 7, 7, 8, 8, 9, 9, 9) separately.

#### 2.1.2. Objective

At the *i*-th image scale, we train generator $G_{i}$ in the manner of adversarial learning to obtain realistic images. It is done by learning $G_{i}$ to minimize the reconstruction loss $\ell_{rec}$ and the adversarial loss $\ell_{adv}$ simultaneously, thereby fooling the discriminator $D_{i}$ to maximize the probability of generated image being classified as real image. Therefore, our objective for optimizing $G_{i}$ is:

(4) $$\min_{G_{i}}\ell_{adv}\left(D_{i}\left(C_{i},G_{i}\left(C_{i},O_{i-1}\right)\right),1\right)+\ell_{rec}\left(C_{i},G_{i}\left(C_{i},O_{i-1}\right),X_{i}\right)$$

and the objective for optimizing $D_{i}$ is

(5) $$\min_{D_{i}}\frac{1}{2}\left(\ell_{adv}\left(D_{i}\left(C_{i},G_{i}\left(C_{i},O_{i-1}\right)\right),0\right)+\ell_{adv}\left(D_{i}\left(C_{i},X_{i}\right),1\right)\right)$$

The same adversarial loss $\ell_{adv}$ as pix2pix [26] is adopted in our implementation. In addition, we propose a mixed reconstruction loss, including the weighted pixel-level loss (WPPL) $\ell_{WPPL}$, multi-scale feature-level VGG [33] loss (MS-FVL) $\ell_{MS-FVL}$, multi-scale feature-level UNet [31] loss (MS-FUL) $\ell_{MS-FUL}$, and multi-scale structural similarity (MS-SSIM) loss [34,35] $\ell_{MS-SSIM}$:

(6) $$\ell_{rec}=\lambda_{WPPL}\times \ell_{WPPL}+\lambda_{MS-SSIM}\times \ell_{MS-SSIM}+\lambda_{MS-FVL}\times \ell_{MS-FVL}+\lambda_{MS-FUL}\times \ell_{MS-FUL}$$

where $\lambda_{WPPL}$, $\lambda_{MS-SSIM}$, $\lambda_{MS-FVL}$, and $\lambda_{MS-FUL}$ denote the loss weights of WPPL, MS-SSIM, MS-FVL, and MS-FUL, respectively. Such a mixed reconstruction loss is able to provide rich and stable gradient information for optimizing generators.

**WPPL.** WPPL computes the weighted mean of L1 distances between the pixels of generated image and real image, where the weight of each pixel is determined by its category, i.e., background, lung, or COVID-19 infection,

(7) $$\ell_{WPPL}\left(C_{i},O_{i},X_{i}\right)=\frac{1}{P}\sum_{p=1}^{P}M\left(C_{i}^{p}\right)\left|O_{i}^{p}-X_{i}^{p}\right|$$

where *p* is the pixel index, *P* is the total number of pixels, and M denotes a mapping function from category to weight. We use L1 loss rather than mean squared error (MSE) loss because optimizing MSE loss tends to obtain over-smoothed image details.

**MS-SSIM loss.** Different from mean-based metrics like L1 distance and MSE, SSIM [36] and MS-SSIM [34] are perceptually motivated metrics to evaluate image similarity based on local structure. As discussed in [35], MS-SSIM loss is differentiated and thus can be back-propagated to optimize CoSinGAN’s parameters. We adopt MS-SSIM loss [35] to optimize the reconstruction of local anatomical structures.

**MS-FVL.** The distance between deep features of two images from a pre-trained CNN classifier is frequently used as the perceptual loss [37,38,39] in image restoration tasks. Compared with pixel-level metrics, perceptual loss is able to obtain visually appealing results. The multi-scale feature-level VGG loss [39] used at the *i*-th scale of CoSinGAN is formulated as:

(8) $$\ell_{MS-FVL}\left(O_{i},X_{i}\right)=\sum_{j=1}^{J}\eta_{j}\frac{1}{P_{j}}{\left\|F_{j}\left(X_{i}\right)-F_{j}\left(G_{i}\left(C_{i},O_{i-1}\right)\right)\right\|}^{1}$$

where $F_{j}$ denotes the *j*-th layer with $P_{j}$ elements of the VGG network [33] and $\eta_{j}$ denotes the weight of the *j*-th feature scale.

**MS-FUL.** Similar with MS-FVL, we design a multi-scale feature-level U-Net loss, which measures the similarity of two images using the deep features from a 2D U-Net [31]:

(9) $$\ell_{MS-FUL}\left(O_{i},X_{i}\right)=\sum_{k=1}^{K}\gamma_{k}\frac{1}{P_{k}}{\left\|F_{k}\left(X_{i}\right)-F_{k}\left(G_{i}\left(C_{i},O_{i-1}\right)\right)\right\|}^{1}$$

where $F_{k}$ denotes the *k*-th layer with $P_{k}$ elements of the 2D U-Net network [31] and $\gamma_{k}$ denotes the weight of *k*-th feature scale. Compared to VGG features that are trained for classification tasks, the 2D U-Net features trained for semantic segmentation encode much more positional and structural information, and thus are more sensitive to the positional distribution of pixels.

#### 2.1.3. Hierarchical Data Augmentation

**Figure 6:**
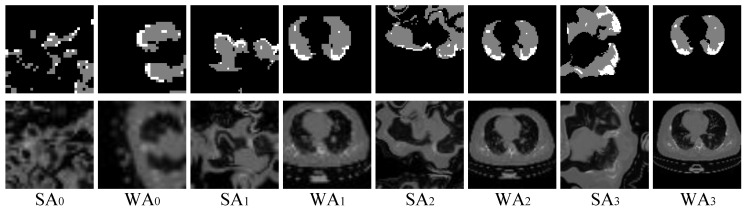
Illustration of augmented images and conditions produced by the hierarchical data augmentation module. SA and WA denote strong augmentation and weak augmentation, respectively. The subscript represents the index of image scale (images are resized for better visualization). The white and gray in condition masks represent the infected regions and lungs, respectively.

As described in Section 2.1.1, to learn the conditional distribution from one single image, one needs to handle the two things well: (a) ensure the generator can generalize to different input conditions, and (b) fit the single image as much as possible for visually accurate and appealing results. Performing strong data augmentation on the single training image is an effective approach to avoid overfitting, whereas it may corrupt the real data distribution and put an additional learning burden on the generator, thus leading to large fitting errors. To address this problem, we propose a hierarchical data augmentation module, involving strong augmentation and weak augmentation, to collaborate with the proposed two-stage GANs at multiple image scales. Specifically, at the *i*-th image scale, the hierarchical data augmentation module produces strong augmentation (SA) to train $G_{is}$, and produces weak augmentation (WA) to train $G_{ir}$. Meanwhile, as the image scale increases, the intensity of SA decreases gradually, whereas WA keeps unchanged. Several augmented images and conditions produced by the hierarchical data augmentation module are illustrated in Figure 6.

The hierarchical data augmentation module has the following advantages:

1. SA is critical for $G_{is}$ to generalize to different input conditions.
2. WA helps to fit the real image distribution without introducing additional learning burden.
3. Decreasing the intensity of SA along with the increasing of image scales can handle the balance between fitting conditions and fitting images well.

Specifically, we implement the hierarchical data augmentation module based on random cropping, random rotation, random horizontal flipping, random vertical flipping, and elastic transform. SA is designed by composing all these transforms, where the cropping size is between 0.5 and 1 times the image size and the parameters of elastic transform is set according to the specific image size. In comparison, WA does not use elastic transform, and the cropping size is between 0.75 and 1 times the image size. Note that the augmentation imposed on images should be consistent with augmentation imposed on conditions all the time. At the *i*-th image scale, to obtain the input of generator $G_{i}$, we perform SA or WA on the original condition and then use the augmented conditions to generate $O_{i-1}$ from scale 0 to scale *i*−1 rather than directly imposing SA or WA on $O_{i-1}$ that is generated by using the original conditions. To make the training converge stably and quickly, we do mini-batch training by taking multiple augmented samples as input.

### 2.2. Materials

We use the two publicly available datasets, i.e., COVID-19-CT-Seg dataset [19] and MosMed dataset [40]. COVID-19-CT-Seg consists of 20 public COVID-19 CT scans with pixel-level annotations of the left lung, right lung, and COVID-19 infection. The annotations, first labeled by junior annotators, are refined by two radiologists with five years of experience, and are further verified and refined by a senior radiologist with more than 10 years of experience in chest radiology. MosMed contains 50 CT scans with only pixel-level annotations of COVID-19 infection (No lung annotations). These 50 CT scans are annotated by the experts of Research and Practical Clinical Center for Diagnostics and Telemedicine Technologies of the Moscow Health Care Department.

We obtain 3520 axial CT slices and 3520 annotation masks of the lungs and COVID-19 infection from the 20 labeled CT volumes in the COVID-19-CT-Seg dataset, and then resize them to 512 × 512. For qualitative analysis, the CT values are normalized directly to [0, 255] before training, whereas, for qualitative analysis, the CT values are first adjusted to lung window [−1250, 250] and then normalized to [0, 255] as done by the COVID-19-CT-Seg-Benchmark. For each CT volume in the COVID-19-CT-Seg dataset, we select one axial slice from it to train a CoSinGAN, and thus get 20 trained CoSinGANs. By taking these 3520 annotation masks as input conditions, each of these models generates 3520 axial CT slices. According to the metadata headers of these 20 real CT volumes, we resize the corresponding synthetic images and stack them in the axial direction to build synthetic CT volumes for further evaluation. Following the experiment setting of learning with limited annotations in COVID-19-CT-Seg-Benchmark, we perform 5-fold cross validation on the COVID-19-CT-Seg data, and independent testing on the MosMed dataset.

## 3. Experiment Results and Discussion

### 3.1. Experiments on Synthesizing Radiological Images

We first conduct ablation experiments on the three key components of CoSinGAN to verify their efficacies. Second, we evaluate the image quality of synthetic radiological images. We finally test the CoSinGAN’s ability in generating diverse samples.

#### 3.1.1. Training Details

We train CoSinGAN with nine image scales, including 32 × 32, 48 × 48, 64 × 64, 96 × 96, 128 × 128, 192 × 192, 256 × 256, 384 × 384 and 512 × 512, which means that nine two-stage GANs are required to train sequentially from coarsest scale to finest scale. Note that we train one stage of a GAN at a time, whereas the other GANs are fixed. The loss weights of WPPL, MS-SSIM, MS-FVL, and MS-FUL in proposed mixed reconstruction loss are empirically set to 10.0, 1.0, 10.0, and 10.0. We set the category weights of background, lung, and COVID-19 infection in WPPL to 0.1, 0.5, and 1.0, respectively, to emphasize the reconstruction of the lungs and infected regions. The VGG network used by MS-FVL is pre-trained on ImageNet, and the 2D U-Net used by MS-FUL is pre-trained on th NSCLC dataset [41] for lung segmentation. Meanwhile, we set the pixel values of these three categories in the input conditions to 0, 128, and 255, respectively. We do strong augmentation to train these two-stage GANs with 4000 epochs and a mini-batch of 4 in their first stage by using an Adam optimizer with the parameters of $\beta_{1}=0.5$ and $\beta_{2}=0.999$. We use an initial learning rate of 0.0002 that is linearly decayed by 0.05% each epoch after 2000 epochs. Correspondingly, we perform weak augmentation to train these two-stage GANs with 2000 epochs and mini-batch of 4 in their second stage by using Adam optimizer with the parameters of $\beta_{1}=0.5$ and $\beta_{2}=0.999$. We use an initial learning rate of 0.0001 that is linearly decayed by 0.1% each epoch after 1000 epochs. All models are trained and evaluated on a linux server equipped with a NVIDIA GeForce GTX 1080 Ti Graphics card.

#### 3.1.2. Ablation Experiments

**Figure 7:**
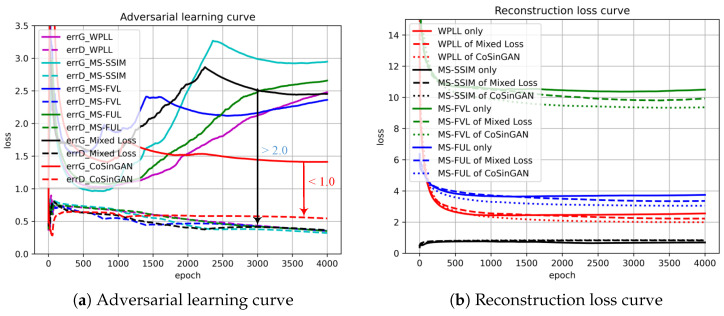
The learning curves of CoSinGAN trained with different reconstruction loss functions at the scale of 256 × 256. We plot the moving average of loss values for better visualization.

**Figure 8:**
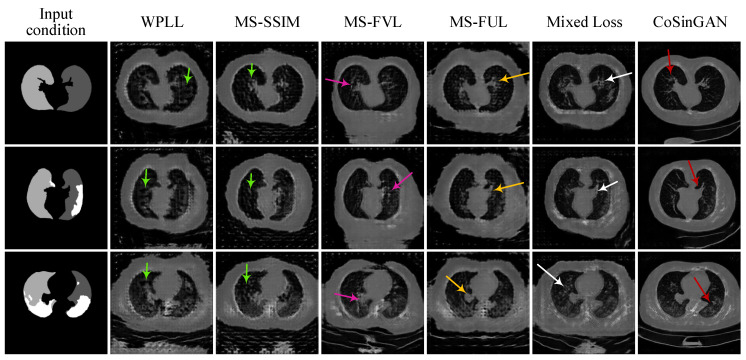
Comparison of synthetic radiological images with image size of 256 × 256 from multiple CoSinGANs trained with different reconstruction loss. Arrows are used to highlight the differences. The white and gray (e.g., light gray and dark gray) in condition masks represent the infected regions and lungs, respectively.

**Mixed reconstruction loss.** We introduce mixed reconstruction loss to provide rich and stable gradient information for optimizing generators. To evaluate its efficacy, we train multiple CoSinGANs at a single image scale of 256 × 256 by using WPLL, MS-SSIM loss, MS-FVL, MS-FVL, and mixed reconstruction loss as the reconstruction loss function separately. The training curves, including adversarial learning curves and reconstruction loss curves, are depicted in Figure 7. The synthetic radiological images from these trained models with given input conditions are shown in Figure 8.

As can be seen, WPLL and MS-SSIM loss, using raw pixel features, synthesizes rich but blurry image details as pointed by green arrows in the 1st and 2nd columns of Figure 8. In comparison, MS-FVL, using deep features of a VGG network pre-trained for image classification, produces visually pleasing images with less noise at the cost of losing local image details as indicated by pink arrows in the fourth column of Figure 8. Compared with MS-FVL, MS-FUL achieves a similar overall visual effect, but produce more image details, e.g., sharp contours and edges (highlighted by yellow arrows in the 4th column of Figure 8). This is probably because the deep features of 2D U-Net used by MS-FUL are trained for lung segmentation and thus encode much more positional and structural information than the deep features of the VGG network. By combining WPLL, MS-SSIM loss, MS-FVL, and MS-FUL, our mixed reconstruction loss can inherit their advantages, and produce visual pleasing images with less noise and more local image details (highlighted by white arrows in the 6th column of Figure 8). Moreover, as illustrated in Figure 7b, the model trained with mixed reconstruction loss achieves consistently smaller WPLL, MS-FVL, and MS-FUL than the same model trained with only one of these loss items. Such results confirm the efficacy of our mixed reconstruction loss in reducing fitting errors.

**Figure 9:**
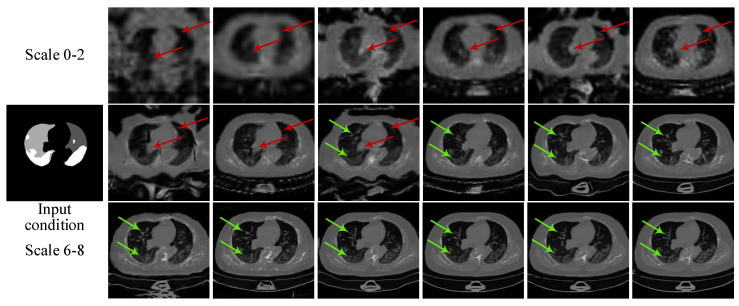
Illustration of the synthesized images with all nine scales from CoSinGAN. Each scale consists of two synthesized images, where one is from the first stage and the other one is from the second stage. All images are resized to 512 × 512 for better visualization. The white and gray (e.g., light gray and dark gray) in condition masks represent the infected regions and lungs, respectively. Red arrows are used to track the contour of the lungs and green arrows are used to track the details of the lungs and infected regions.

**Multi-scale architecture and two-stage GAN.** We train a complete CoSinGAN with all nine image scales on a single radiological image. We plot the adversarial learning curve and reconstruction loss curve of CoSinGAN at the image scale of 256 × 256 in Figure 7. As can be seen from Figure 7a, the complete CoSinGAN has a better adversarial learning curve than the other models trained with only a single image scale. The adversarial loss values of generator *G* and discriminator *D* are close to each other throughout the entire training process. It indicates that the adversarial training of CoSinGAN is stable and thus *G* is able to capture the distribution of real images gradually through continuously fighting with *D*. The reconstruction loss curve in Figure 7b also shows that CoSinGAN trained with multi-scale architectures achieves lower fitting error. In addition, as illustrated in Figure 8, the radiological images produced by the complete CoSinGAN present significantly better visual effects with realistic and sharp image details (highlighted by red arrows in the last column). Such results strongly verify the effectiveness of multi-scale architecture. Moreover, we use the complete CoSinGAN to generate images with all nine scales and compare them in Figure 9. Each scale includes two synthetic images, where one is from the first stage and the other one is from the second stage. We use the red arrows to track the contour of the lungs to highlight the efficacy of proposed multi-scale architecture and two-stage GAN in enhancing the condition constraints. We notice that the synthetic images from the first stage match the input condition much better than the images from the second stage at scale 0–2, whereas the images from the second stage are more realistic and clear than the images from the first stage. As the image scale increases (scale 6–8), the synthetic images from both stages are able to match the input condition gradually and precisely. In addition, we use the green arrows to track the details of the lungs and infected regions in synthetic images as the image scale increases. Intuitively, the image details are enhanced progressively. Such results strongly confirm that the multi-scale architectures are able to collaborate with the two-stage GANs by iteratively enhancing condition constraints and image details.

**Hierarchical data augmentation.** As illustrated in Figure 6, our hierarchical data augmentation module is able to produce strong augmentation (SA) for the first stage of GAN and weak augmentation (WA) for the second stage of GAN. SA is designed to enhance conditions, while WA is used to facilitate the restoration of image details. As can be seen from the first three scales of synthesized images in Figure 9, the contours of the lungs in the synthetic images from the first stage generators are maintained better than those from the second stage generators. It presents that the first stage generators trained with SA present a stronger generalization ability to input conditions than the second stage generators trained with WA. In addition, the synthetic images from the second stage tend to be more realistic and contain more image details than those from the first stage. As the image scale increases, the intensity of SA gradually decreases, whereas the synthetic images match the input condition better and better. Benefiting from the output from previous scales, the generators in the later scales trained with relatively weaker SA are still able to generalize to input conditions. Thus, more gradients can be assigned to optimize image details. Such results clearly confirm that our hierarchical data augmentation module is able to provide a well balance between preventing the generators from being overfitted to input condition and overfitting the single training image as much as possible when learning conditional distribution from a single image.

#### 3.1.3. Evaluation and Comparison on Image Quality

**Baselines**. The pix2pix model [26] is a well-known conditional GAN framework for image-to-image translation. In our implementation, we design an enhanced pix2pix model by replacing L1 reconstruction loss in the pix2pix model with the proposed mixed reconstruction loss to build our two-stage GAN. Accordingly, the pix2pix model and the enhanced pix2pix model are used as the baseline models in our experiments to highlight our contributions. Note that the baseline pix2pix model is also implemented with the weighted L1 reconstruction loss, i.e., WPLL, to emphasize the reconstruction of the lungs and infected regions.

**Figure 10:**
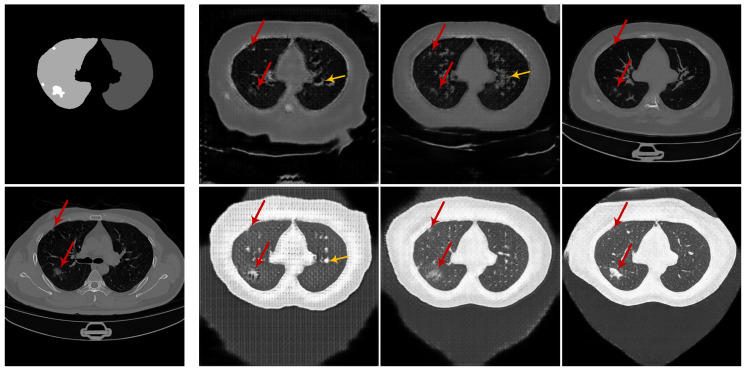
Comparison of synthetic radiological images from different generative models given the same input condition. The input condition and the reference ground-truth radiological image are depicted in the first column. The last three columns are the results of pix2pix, enhanced pix2pix, and CoSinGAN, respectively. Red arrows are used to track and highlight the small infected regions, and yellow arrows are used to indicate the artifacts. The white and gray (e.g., light gray and dark gray) in condition masks represent the infected regions and lungs, respectively.

**Figure 11:**
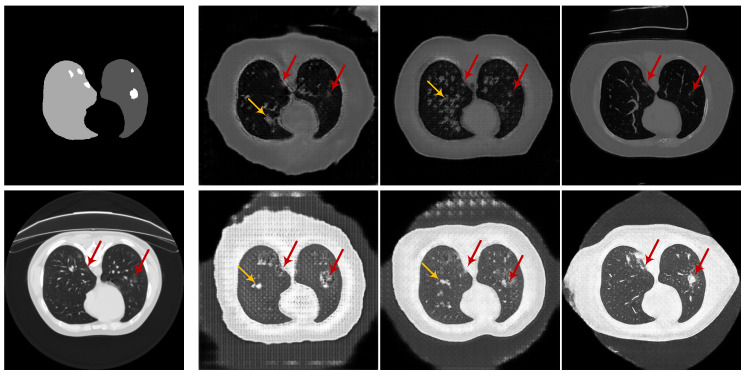
Comparison of synthetic radiological images from different generative models given the same input condition. The input condition and the reference ground-truth radiological image are depicted in the first column. The last three columns are the results of pix2pix, enhanced pix2pix, and CoSinGAN, respectively. Red arrows are used to track and highlight the small infected regions, and yellow arrows are used to indicate the artifacts. The white and gray (e.g., light gray and dark gray) in condition masks represent the infected regions and lungs, respectively.

**Qualitative comparison.** The synthetic radiological images from the two trained CoSinGANs are illustrated in Figure 3 and Figure 4. As can be seen, our CoSinGAN is very sensitive to the input conditions as even the small isolated infected regions are reconstructed precisely (indicated by red circles). Meanwhile, these synthetic radiological images are able to present sharp and rich image details with less noise and clean background, comparable to the real radiological image with the size of 512 × 512. The visual features of the lungs and infected regions are also synthesized consistently with the single training image. Such ability of CoSinGAN is critical for building the synthetic training samples with pixel-level annotations of the lungs and COVID-19 infection to learn lung and COVID-19 infection segmentation. Next, given the same input conditions, we compare the results of different generative models in Figure 10 and Figure 11. These input conditions are sampled from different labeled CT scans, where their relative real CT slices are called reference ground-truth images. As can be seen, our CoSinGAN can produce visually appealing results with clear image details and clean background, which are significantly better than the results of the baseline models and comparable to the reference ground-truth images. The synthetic images from the pix2pix model contain too many visually unpleasant artifacts, which make it tough to distinguish the infected regions from these artifacts (highlighted by yellow arrows). With the help of the mixed reconstruction loss, the enhanced pix2pix model achieves a better visual effect with fewer grid artifacts and richer lung details in comparison of pix2pix. Despite that, the synthesized details of lungs are still not clear enough to be distinguished from the infected regions (indicated by yellow arrows in the third column). Such synthetic images from the pix2pix model and the enhanced pix2pix model may not be used to learn lung and COVID-19 infection segmentation smoothly. In comparison, our CoSinGAN effectively solves the problems of grid artifacts and blurry lung details, and can produce high-quality radiological images with pixel-level annotations for training deep segmentation models.

**Quantitative comparison.** The synthetic radiological images are used to train deep models for automated lung and infection segmentation. Therefore, we directly adopt the task-driven evaluation method to quantify the image quality. Specifically, we train 2D U-Net [32] on these synthetic radiological images to perform lung and infection segmentation on real radiological images, and take the segmentation performance as a criterion to measure the image quality and compare different generative models. Following the setting of the COVID-19-CT-Seg-Benchmark, the CT cases in the COVID-19-CT-Seg dataset are first adjusted to lung window [−1250, 250] and then normalized to [0, 255] before training generative models. The nnU-Net (no-new-U-Net) [32], one of the state-of-the-art implementation of U-Net [31], is adopted as our segmentation model. We use the pre-defined train-validation splits provided by COVID-19-CT-Seg-Benchmark to perform 5-fold cross validation. In each fold, 80 synthetic CT cases, corresponding to 4 CT slices from four real cases in COVID-19-CT-Seg dataset, are used for training, and the rest 16 real cases are used for testing. We train the segmentation model with 100 epochs, and all other training configurations are the default. Normalized surface Dice (NSD) [42] and Dice similarity coefficient (DSC) are adopted as the segmentation evaluation metrics. A higher score for both two metrics indicates better segmentation performance, and further admits higher image quality. The image quality (segmentation) scores are reported in Table 1.

As can be seen, our CoSinGAN obtains the highest average segmentation scores on both lung and COVID-19 infection, surpassing the baseline methods by a large margin. It indicates that CoSinGAN can reconstruct the lungs and infected regions more precisely at the locations depicted by the input conditions. Such results strongly confirm the efficacy of our CoSinGAN in learning the conditional distribution of radiological image from a single radiological image. In addition, we notice that the enhanced pix2pix model using our mixed reconstruction loss achieves much higher segmentation scores than the pix2pix model, which demonstrates the effectiveness of our mixed reconstruction loss in improving image quality.

#### 3.1.4. Evaluation on the Ability of CoSinGAN in Generating Diverse Samples

Given an input condition, we expect that CoSinGAN can generate diverse samples, which are different but correlated in visual appearance. We explore three approaches to make CoSinGAN capable of generating diverse samples, including applying dropout at test time, randomizing the input condition and fusing synthetic images from two CoSinGANs. We call them data diversification methods. We expect to use these methods to improve the segmentation performance of deep models trained on synthetic samples.

**Figure 12:**
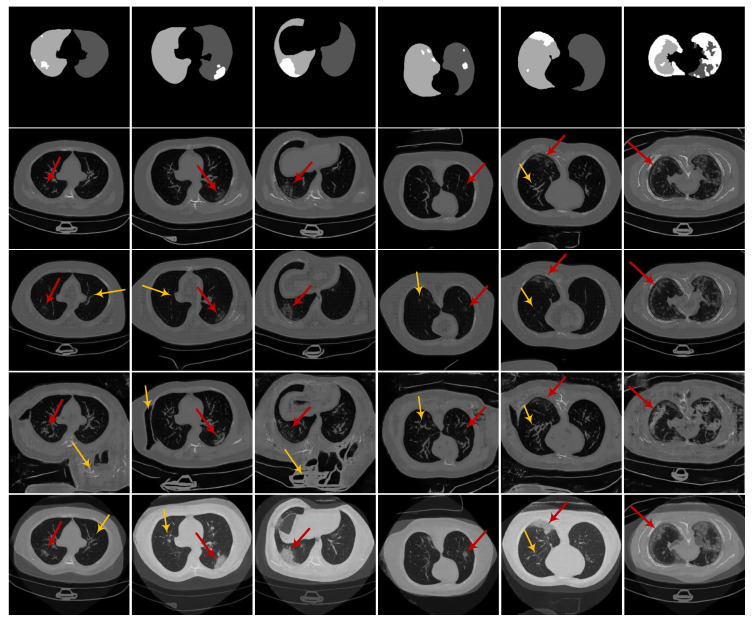
Comparison of diverse synthetic images from CoSinGAN by using different data diversification methods. The rows from top to bottom represent input conditions, synthetic images without using any data diversification methods, results of applying dropout at test time, results of randomizing input condition, and results of fusing synthetic images from two different CoSinGANs, respectively. Red and yellow arrows highlight the differences of synthetic images. The white and gray (e.g., light gray and dark gray) in condition masks represent the infected regions and lungs, respectively.

**Applying dropout (AD).** Applying dropout at inference time with a dropout rate of 50% can add randomness to the forward propagation of CoSinGAN through randomly inactivating some activation units of neural network. As shown in Figure 12, this operation has a slight effect on CoSinGAN’s output, such as the weakening of local image details (indicated by arrows in the 3-th row). Thus, this approach may not have much effect on the diversity of synthetic radiological images.

**Randomizing input condition (RC).** During the training process, the pixel values of background, lung and COVID-19 infection in the input conditions are set to 0, 128, and 255, respectively. After training, we can randomize the input condition by adding random noise to it to synthesize diverse images. Specifically, the pixel values of background, lung, and COVID-19 infection are randomly set as follows:

(10) $$V_{background}\in \left[0,\delta_{b}\right],V_{lung}\in \left[128-\delta_{l},128+\delta_{l}\right],V_{infection}\in \left[255-\delta_{i},255\right]$$

where $\delta_{b}$, $\delta_{l}$, and $\delta_{i}$ denote the magnitudes of the random noises. In our experiment, we set them to 16, 16, and 32, respectively. It is worth noting that such randomness is applied in every image scale. This means that the input condition at each image scale may be different, which further promotes the diversity of synthetic images. As shown in Figure 12, RC produces diverse radiological images with notable differences in background, local lung details, and COVID-19 infection (highlighted by the arrows in the 4th row). In addition, RC does not damage the sharpness and local details of synthetic images. Although these synthetic images appear to be lack of clinical evidence, such results confirm that the RC is an effective data diversification method, which may improve the generalization ability of segmentation model trained on synthetic samples.

**Image fusion (IF).** We propose to fuse the synthetic images from two different CoSinGANs that are trained separately on two different single images. Given the same input condition, the two CoSinGANs can generate paired images that have different but correlated visual appearance and match each other pixel-by-pixel. Accordingly, we can simply fuse the two paired images without losing image details as follows:

(11) $$I_{f}=\zeta \times I_{1}+\left(1-\zeta \right)\times I_{2}$$

where ζ is the fusion coefficient. We introduce diversity by randomly setting the value of ζ from 0.0 to 1.0 in our experiments. As shown in the last row of Figure 12, IF can produce radiological images with different visual appearances. This method is similar with the mixup operation [43], which is commonly used as a data augmentation method. Compared with mixup, the IF method, fusing two paired images, is more likely to obtain accurate visual features of the lungs and infected regions, and thus may help to improve the segmentation performance of deep model trained on synthetic samples.

### 3.2. Experiments on Learning Deep Models for Automated Lung and Infection Segmentation

**Figure 13:**
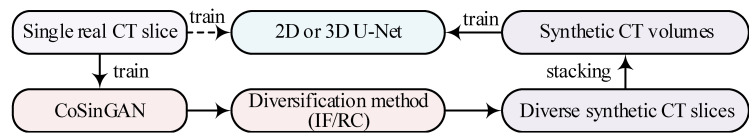
Illustration of the workflow of learning 2D or 3D U-Net from a single CT slice for automated lung and infection segmentation by using CoSinGAN.

The workflow of learning deep models for automated lung and infection segmentation from a single radiological image by using our CoSinGAN is illustrated in Figure 13.

#### 3.2.1. Baselines

We use the COVID-19-CT-Seg-Benchmark [19] as our baseline. The benchmark is designed to solve the problem of few-short learning, where few annotations are available for training. A pre-defined train-validation split file is provided by COVID-19-CT-Seg-Benchmark to perform 5-fold cross validation on COVID-19-CT-Seg dataset and independent testing on the MosMed dataset. In each fold, 4 of 20 CT cases in COVID-19-CT-Seg dataset are used for training, and the rest of the 16 CT cases in COVID-19-CT-Seg dataset and 50 CT cases in MosMed dataset are used for testing as shown in Table 2. The average number of labeled axial CT slices used for training is 704. Considering that our method uses only four labeled CT slices, the benchmark is quite a strong baseline. Both the 2D U-Net baseline and 3D U-Net baseline are available in the COVID-19-CT-Seg-Benchmark. Note that the MosMed dataset provides infection annotations only and thus we only report the infection segmentation results for it. In contrast, for the COVID-19-CT-Seg dataset, we report the left lung, right lung, and infection segmentation results.

In addition, learning COVID-19 infection segmentation from off-the-shelf non-COVID-19 CT scans (e.g., lung cancer and pleural effusion) is a natural solution at the early stage of COVID-19 outbreak. Therefore, we also compare CoSinGAN with such solutions provided by COVID-19-CT-Seg-Benchmark. Specifically, COVID-19-CT-Seg-Benchmark has attempted to use the MSD Lung Tumor dataset (63 lung tumor CT scans) [44], the StructSeg2019 dataset (50 lung tumor CT scans) [45], and the NSCLC Pleural Effusion dataset (78 pleural effusion CT scans) [41] separately with or without COVID-19 CT scans to learn automated COVID-19 infection segmentation. The experiment settings are detailed in Table 3 and Table 4. More details can be found in [19] (https://gitee.com/junma11/COVID-19-CT-Seg-Benchmark).

#### 3.2.2. CoSinGAN, IF-CoSinGAN, and RC-CoSinGAN

To emphasize the effectiveness of our method in learning deep models from few radiological images for lung and infection segmentation, we following the setting of the benchmark expect for using only four labeled CT slices, which are extremely less than the average of 704 labeled CT slices used by the benchmark. IF-CoSinGAN and RC-CoSinGAN denote CoSinGANs that use the IF and RC methods, respectively. We use the same train-validation splits to perform 5-fold cross validation. In each fold, 80 synthetic CT cases, corresponding to four CT slices from four real cases in the COVID-19-CT-Seg dataset, are used for training, and the rest of the 16 CT cases in COVID-19-CT-Seg dataset and 50 CT cases in MosMed dataset are used for testing as shown in Table 2. In this way, we build three synthetic training datasets corresponding to CoSinGAN, IF-CoSinGAN, and RC-CoSinGAN, respectively.

#### 3.2.3. Segmentation Models and Training Details

As with COVID-19-CT-Seg-Benchmark, we use the nnU-Net (no-new-U-Net) [40] to perform left lung, right lung, and COVID-19 infection segmentation. In particular, nnU-Net is one of the state-of-the-art implementation of U-Net, and can adapt preprocessing strategies (e.g., data augmentation operations) and network architectures (e.g., the size of convolutional kernel) automatically to a given 3D medical dataset. Our CoSinGAN is designed to synthesize 2D radiological images, thus we only train 2D U-Net on these three synthetic training datasets separately to compare with the 2D U-Net baseline. Typically, 3D U-Net is more competitive than 2D U-Net in 3D medical segmentation tasks. Learning a 3D U-Net from few 2D slices is very challenging but meaningful. In addition, we are also interested in the performance of 3D U-Net trained on synthetic 3D volumes that are obtained by stacking synthetic 2D CT slices from CoSinGAN. Therefore, we simply train 3D U-Net on the synthetic training dataset of CoSinGAN to test the ability of the synthetic 2D radiological images in learning 3D segmentation models by comparing with the 3D U-Net baseline. We train these segmentation models on each synthetic dataset respectively by using the same default setting of nnU-Net as COVID-19-CT-Seg-Benchmark.

#### 3.2.4. Evaluation Metrics

As with COVID-19-CT-Seg-Benchmark, two evaluation metrics are adopted, including normalized surface Dice (NSD) and Dice similarity coefficient (DSC). NSD [42] is a boundary-based measure to evaluate how close the segmentation results and the ground-truth surfaces are to each other at a specified tolerance. DSC is a region-based measure to evaluate the region overlap. A higher score for both two metrics indicates better segmentation performance. In our experiment, we directly use the evaluation tools provided by COVID-19-CT-Seg-Benchmark to compute the two metrics.

#### 3.2.5. Results and Discussion

(**1**) **Quantitative comparison**

**CoSinGAN versus COVID-19-CT-Seg-Benchmark based on 2D U-Net.** The quantitative results of 5-fold cross validation and independent testing based on 2D U-Net are reported in Table 5. First, we compare CoSinGAN with the benchmark. On the COVID-19-CT-Seg dataset, CoSinGAN achieves notable lung segmentation scores, i.e., average DSC: 93% and average NSD: 76%, which are much higher (more than 4%) than the benchmark results. More importantly, the COVID-19 infection segmentation scores of CoSinGAN are also superior on both COVID-19-CT-Seg dataset and MosMed dataset (50 CT cases). Note that our CoSinGAN uses only four real CT slices, whereas the benchmark uses an average of 704 real CT slices. Such results strongly confirm the efficacy of our method in learning automated lung and infection segmentation from very few labeled CT slices. Next, we compare IF-CoSinGAN and RC-CoSinGAN with CoSinGAN. As shown in Table 5, IF-CoSinGAN and RC-CoSinGAN achieve very close lung segmentation scores to CoSinGAN, which indicates the diversification methods, i.e., IF and RC, does not improve the lung segmentation. In addition, we notice that the infection segmentation scores of IF-CoSinGAN on MosMed datasets are notably lower than CoSinGAN. It shows that the IF method does not work as we expect. This is probably because too many fused infected regions make the distribution of synthetic images deviate from that of real images. One way to improve it might be tuning the distribution of fusion coefficient ζ in (11), for instance, choosing a random ζ from a Beta distribution as the mixup method [43] does. In contrast, RC-CoSinGAN surpasses CoSinGAN by 7.2% of DSC and 11.5% of NSD on COVID-19-CT-Seg dataset, and by 5.0% of DSC and 4.8% of NSD on the MosMed dataset, respectively. It implies that the RC method, randomizing input condition of CoSinGAN, does not make the distribution of diverse synthetic images deviate from that of real images. Instead, RC may produce several new synthetic radiological images that satisfy the distribution of real CT slices but are different with the four training CT slices. Such results confirm that RC is an effective data diversification approach for CoSinGAN, and also demonstrate that our CoSinGAN owns strong ability in learning conditional distribution from a single radiological image. Finally, we compare RC-CoSinGAN with the benchmark. We observe that RC-CoSinGAN surpasses the benchmark in both lung segmentation and infection segmentation by a large margin. Such results are of greater significance when noticing that RC-CoSinGAN uses only four real CT slices, i.e., 1/176 of real CT slices used by the benchmark.

**Figure 14:**
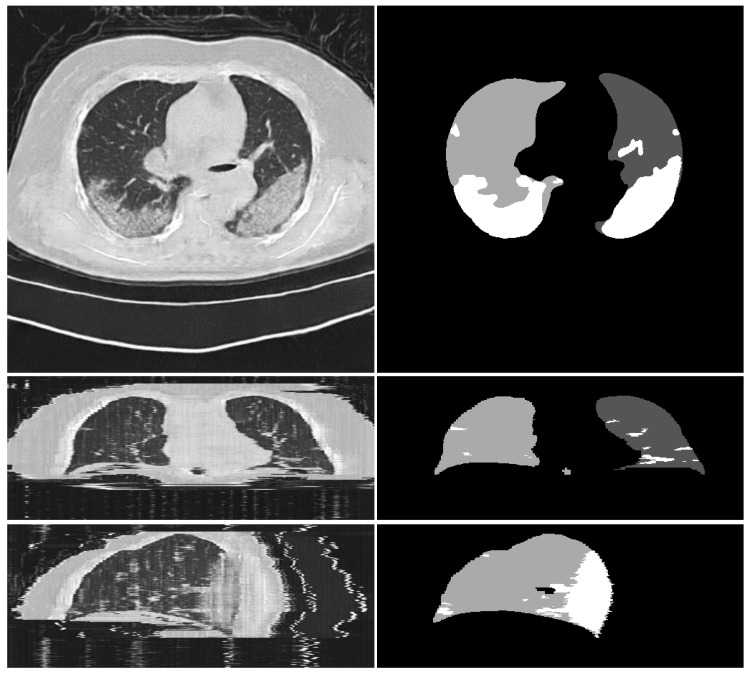
Illustration of the axial, coronal, and sagittal views of a synthetic CT volume. The white and gray (e.g., light gray and dark gray) in condition masks represent the infected regions and lungs, respectively.

**CoSinGAN versus COVID-19-CT-Seg-Benchmark based on 3D U-Net.** On the other hand, we report the quantitative results of 5-fold cross validation and independent testing based on 3D U-Net in Table 6. As can be seen, the lung segmentation scores of CoSinGAN are significantly lower (more than 10%) than the benchmark results. The result is reasonable because our CoSinGAN is designed for synthesizing 2D radiological images rather than 3D volumes. The 3D structures of the lungs cannot be reconstructed accurately by stacking 2D synthetic axial CT slices. We depict the axial, coronal, and sagittal views of a synthetic CT volume in Figure 14 to illustrate such problem. The blurry lung details in the coronal and sagittal views confirm our claims. In contrast, CoSinGAN achieves notable infection segmentation scores, which are comparable (less than 1%) to the benchmark results on the COVID-19-CT-Seg dataset and are superior (more than 3%) to the benchmark results on the MosMed dataset. Compared with the lungs, the infected region has no fixed shape, size, or 3D context, and its visual features are much simpler. Thus, the synthetic CT volumes work well for learning 3D infection segmentation.

**CoSinGAN versus learning with non-COVID-19 CT scans in the COVID-19-CT-Seg-Benchmark based on 2D U-Net.** We report the quantitative results of different methods for learning automated COVID-19 infection segmentation based on 2D U-Net in Table 7. As can be seen, our methods, learning from very few CT slices by synthesizing diverse CT slices, have achieved significantly better COVID-19 infection segmentation performance than the other methods that learn from non-COVID-19 CT scans. We notice that the gains in COVID-19 infection segmentation performance from learning with other lung lesion CT scans (e.g., lung cancer and pleural effusion) are rather limited, or, to be more precise, are negative. Although the use of non-COVID-19 CT scans can increase the variety of training samples, it may make the distribution of training samples deviate from that of real COVID-19 CT slices due to the domain gap between non-COVID-19 CT scans and COVID-19 CT scans. One way to improve it might be adopting unsupervised domain adaptation, which is far beyond the scope of this paper.

(**2**) **Qualitative comparison**

**Figure 15:**
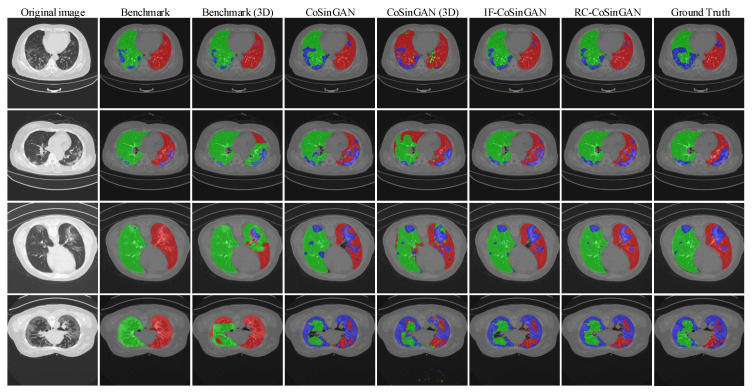
Visual comparison of lung and infection segmentation results on the COVID-19-CT-Seg dataset, where the red, green, and blue represent the left lung, right lung, and infected regions, respectively. Benchmark (3D) and CoSinGAN (3D) represent their results are based on 3D U-Net, whereas the others are all based on 2D U-Net. All results here come from the Fold-4. The blue, green, and red denote the infected regions, right lung and left lung, respectively.

**Figure 16:**
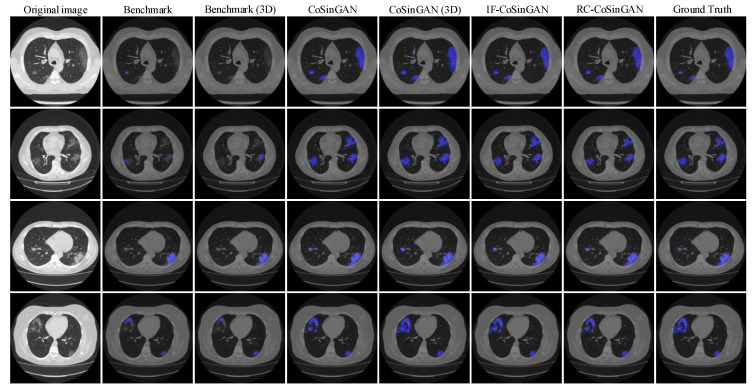
Visual comparison of infection segmentation results on the MosMed dataset, where the blue represents infected regions. Benchmark (3D) and CoSinGAN (3D) represent their results are based on 3D U-Net, whereas the others are all based on 2D U-Net. All results here come from the Fold-4. The blue denotes the infected regions.

Finally, we illustrate the lung and infection segmentation results on the COVID-19-CT-Seg dataset in Figure 15 and the infection segmentation results on the MosMed dataset in Figure 16. All results come from the Fold-4. We notice that the segmentation results of our CoSinGANs on both COVID-19-CT-Seg dataset and MosMed datasets are close to the ground-truth with much less inaccurate segmentation. In contrast, the benchmarks yield unsatisfactory results, where a large number of infected regions are not well recognized and segmented. The visual comparison indicates that our CoSinGANs learn lung and infection segmentation well from few CT slices and outperform the benchmarks remarkably. The success of CoSinGAN is owed to its strong ability of learning conditional distribution from a single radiological image and the effective data diversification method. Through the workflow illustrated in Figure 13, our CoSinGAN can learn both 2D and 3D segmentation models from few labeled CT slices and achieve much better segmentation performance than the counterparts that uses the same few labeled CT volumes. Therefore, our CoSinGAN is much more annotation-efficient than the COVID-19-CT-Seg-Benchmark.

## 4. Conclusions

The highly contagious COVID-19 has spread rapidly and overwhelmed healthcare systems across the world. Automated infection measurement and COVID-19 diagnosis at the early stage is critical to prevent the further evolving of COVID-19 pandemic. We propose a novel approach, called CoSinGAN, for learning automated lung and infection segmentation from extremely few radiological images. Our CoSinGAN is able to capture the conditional distribution of the single radiological image, and further synthesize high-resolution (512 × 512) and diverse radiological images that match the input conditions precisely. We evaluate the efficacy of CoSinGAN in learning lung and infection segmentation from very few radiological images by performing 5-fold cross validation on COVID-19-CT-Seg dataset (20 CT cases) and independent testing on the MosMed dataset (50 CT cases). Both 2D U-Net and 3D U-Net, learned from four CT slices by using our CoSinGAN, have achieved notable infection segmentation performance, surpassing the COVID-19-CT-Seg-Benchmark, i.e., the counterparts trained on an average of 704 CT slices, by a large margin. Such results strongly confirm that our method has the potential to realize learning COVID-19 diagnosis from few radiological images. Therefore, the findings of this study provide promising results that encourage the use of our CoSinGAN to build more robust and timely radiology systems at the early stage of COVID-19 outbreak. The limitation of our work is that the structured masks of the lungs and infected regions still need to be drawn manually. In the future, we intend to design an automated mask generator for CoSinGAN to make our approach more scalable. Moreover, we also intend to extend our CoSinGAN to synthesize CT volumes that satisfy the 3D context of the lungs directly to further improve the lung and infection segmentation performance.

## Figures and Tables

**Table 1:** Quantitative results (Mean ± Standard deviation) of 5-fold cross validation based on 2D U-Net. For each fold, average DSC and NSD values are reported. The last row shows the average results of 80 (=5 folds × 16 testing cases per fold) testing cases in a COVID-19-CT-Seg dataset. ↑ denotes a higher score and admits better segmentation performance. Our CoSinGAN surpasses the baseline methods by a large margin. The entries in bold highlight the best average results.

Subtask	Model	Left Lung		Right Lung		Infection (COVID-19-CT-Seg)	
		DSC ↑	NSD ↑	DSC ↑	NSD ↑	DSC ↑	NSD ↑
Fold-0	Pix2pix	91.4 ± 6.8	72.7 ± 11.7	91.7 ± 6.3	69.1 ± 11.4	46.3 ± 19.8	43.8 ± 20.1
	Enhanced pix2pix	86.9 ± 15.4	69.1 ± 16.3	88.9 ± 11.4	68.7 ± 15.8	50.4 ± 22.6	47.3 ± 21.1
	CoSinGAN	93.1 ± 6.3	76.7 ± 11.5	94.5 ± 4.1	76.4 ± 10.3	37.0 ± 21.0	35.6 ± 20.8
Fold-1	Pix2pix	86.0 ± 10.3	60.0 ± 15.3	87.2 ± 10.8	60.2 ± 15.3	42.7 ± 22.7	36.5 ± 23.8
	Enhanced pix2pix	92.6 ± 5.9	74.6 ± 10.5	94.2 ± 3.6	75.3 ± 8.3	60.3 ± 20.3	55.9 ± 24.0
	CoSinGAN	91.9 ± 7.0	74.0 ± 12.1	94.2 ± 3.9	74.6 ± 9.6	60.3 ± 21.1	53.8 ± 23.4
Fold-2	Pix2pix	81.6 ± 11.7	52.2 ± 19.2	80.5 ± 13.4	49.7 ± 17.8	41.2 ± 16.6	39.7 ± 18.0
	Enhanced pix2pix	92.5 ± 4.5	70.7 ± 9.7	93.4 ± 3.3	70.1 ± 7.3	44.0 ± 20.8	43.8 ± 22.5
	CoSinGAN	91.9 ± 6.4	73.0 ± 11.2	92.5 ± 4.8	70.6 ± 8.9	48.1 ± 19.6	45.0 ± 20.3
Fold-3	Pix2pix	83.4 ± 18.6	57.3 ± 18.5	83.7 ± 17.6	54.3 ± 19.6	40.3 ± 22.0	38.7 ± 23.2
	Enhanced pix2pix	92.5 ± 7.0	73.0 ± 12.1	93.4 ± 4.0	69.8 ± 10.4	55.1 ± 19.3	50.6 ± 23.4
	CoSinGAN	94.5 ± 5.2	79.8 ± 9.9	95.3 ± 2.5	78.3 ± 8.6	62.6 ± 20.0	59.3 ± 23.0
Fold-4	Pix2pix	86.2 ± 13.1	59.6 ± 13.7	87.4 ± 9.8	57.9 ± 13.1	43.1 ± 14.9	41.1 ± 16.0
	Enhanced pix2pix	90.1 ± 9.7	69.3 ± 14.0	91.0 ± 6.3	67.1 ± 13.6	48.3 ± 13.9	49.2 ± 18.2
	CoSinGAN	91.8 ± 5.6	71.4 ± 10.3	92.3 ± 6.0	69.8 ± 11.0	62.4 ± 12.6	58.5 ± 15.9
Avg	Pix2pix	85.8 ± 13.1	60.3 ± 17.3	86.1 ± 12.7	58.2 ± 17.0	42.7 ± 19.5	40.0 ± 20.6
	Enhanced pix2pix	90.9 ± 9.6	71.3 ± 12.9	92.2 ± 6.8	70.2 ± 11.9	51.6 ± 20.4	49.4 ± 22.2
	CoSinGAN	**92.6 ± 6.2**	**75.0 ± 11.4**	**93.8 ± 4.6**	**73.9 ± 10.3**	**54.1 ± 21.6**	**50.4 ± 22.7**

**Table 2:** Experiment settings of learning deep models with only COVID-19 CT scans (or slices) for automated lung and infection segmentation.

Methods	COVID-19-CT-Seg Dataset (20 CT Cases)		MosMed Dataset (50 CT Cases)
	Training	Testing	Testing
Strong baselines (Benchmark)	4 CT cases with an averageof 704 CT slices	16 CT cases	50 CT cases
CoSinGAN	4 CT slices → 80 synthetic CT volumes	16 CT cases	50 CT cases
IF-CoSinGAN	4 CT slices → 80 synthetic CT volumes	16 CT cases	50 CT cases
RC-CoSinGAN	4 CT slices → 80 synthetic CT volumes	16 CT cases	50 CT cases

**Table 3:** Experiment settings of learning deep models with only non-COVID-19 CT scans for automated infection segmentation.

Methods	Training	Testing
Task2-MSD	MSD Lung Tumor dataset (51 CT cases)	COVID-19-CT-Seg dataset (20 CT cases)
Task2-StructSeg	StructSeg2019 (40 CT cases)	COVID-19-CT-Seg dataset (20 CT cases)
Task2-NSCLC	NSCLC Pleural Effusion dataset (62 CT cases)	COVID-19-CT-Seg dataset (20 CT cases)

**Table 4:** Experiment settings of learning deep models with both COVID-19 and non-COVID-19 CT scans for automated infection segmentation.

Methods	Training		Testing
Task3-MSD	MSD Lung Tumor dataset (51 CT cases)	COVID-19-CT-Seg dataset (4 CT cases)	COVID-19-CT-Seg dataset (16 CT cases)
Task3-StructSeg	StructSeg2019 (40 CT cases)	COVID-19-CT-Seg dataset (4 CT cases)	COVID-19-CT-Seg dataset (16 CT cases)
Task3-NSCLC	NSCLC Pleural Effusion dataset (62 CT cases)	COVID-19-CT-Seg dataset (4 CT cases)	COVID-19-CT-Seg dataset (16 CT cases)

**Table 5:** Quantitative results (Mean±Standard deviation) of 5-fold cross validation and independent testing based on 2D U-Net. For each fold, average DSC and NSD values are reported. The last row shows the average results of 80 (=5 folds × 16 testing cases per fold) testing cases in the COVID-19-CT-Seg dataset and 250 (=5 folds × 50 testing cases per fold) testing cases in the MosMed dataset. The third column shows the number of real labeled CT slices used for training. ↑ denotes a higher score that admits better segmentation performance. The entries in bold highlight the best average results.

Subtask	Methods	Slice Number	Left Lung		Right Lung		Infection (COVID-19-CT-Seg)		Infection (MosMed)	
			DSC ↑	NSD ↑	DSC ↑	NSD ↑	DSC ↑	NSD ↑	DSC ↑	NSD ↑
Fold-0	Benchmark	634	89.9 ± 10.7	72.8 ± 18.6	91.3 ± 8.6	73.5 ± 18.1	67.8 ± 20.7	69.1 ± 23.0	53.2 ± 20.1	62.5 ± 18.7
	CoSinGAN	4	92.6 ± 7.9	78.4 ± 11.9	93.9 ± 5.1	77.5 ± 10.1	61.9 ± 19.3	55.6 ± 21.2	39.5 ± 24.6	47.5 ± 23.6
	IF-CoSinGAN	4	93.8 ± 5.2	79.0 ± 9.7	94.7 ± 3.8	78.3 ± 9.5	58.7 ± 20.5	54.2 ± 20.4	29.0 ± 20.1	38.1 ± 19.6
	RC-CoSinGAN	4	94.1 ± 5.5	81.8 ± 10.3	95.0 ± 3.8	80.2 ± 8.8	73.9 ± 20.2	73.7 ± 21.8	54.0 ± 22.4	62.0 ± 21.3
Fold-1	Benchmark	681	87.7 ± 13.9	68.6 ± 17.5	90.4 ± 9.3	70.4 ± 14.4	61.5 ± 21.6	58.1 ± 26.8	44.5 ± 19.9	51.4 ± 19.6
	CoSinGAN	4	93.4 ± 4.6	75.9 ± 8.6	94.3 ± 3.2	75.6 ± 8.6	67.9 ± 19.6	62.8 ± 21.8	52.0 ± 21.3	59.6 ± 20.2
	IF-CoSinGAN	4	93.7 ± 4.9	77.1 ± 9.4	94.7 ± 3.4	76.9 ± 8.0	67.1 ± 19.7	61.1 ± 22.2	41.8 ± 21.7	48.6 ± 19.9
	RC-CoSinGAN	4	93.6 ± 4.7	77.8 ± 8.6	94.6 ± 3.2	77.1 ± 7.4	71.3 ± 20.4	70.5 ± 22.5	41.9 ± 22.5	49.5 ± 22.7
Fold-2	Benchmark	683	91.1 ± 12.4	76.0 ± 16.1	92.5 ± 8.8	74.2 ± 15.6	52.5 ± 25.2	49.1 ± 25.0	41.2 ± 22.6	45.2 ± 21.4
	CoSinGAN	4	93.3 ± 5.2	76.2 ± 9.4	94.2 ± 3.7	75.1 ± 8.2	61.7 ± 21.4	59.3 ± 23.0	36.4 ± 22.0	43.2 ± 20.8
	IF-CoSinGAN	4	93.1 ± 5.3	75.5 ± 10.4	93.9 ± 4.1	74.0 ± 9.4	60.8 ± 19.7	56.7 ± 21.9	31.6 ± 22.1	38.4 ± 19.9
	RC-CoSinGAN	4	94.2 ± 5.0	79.3 ± 9.6	94.6 ± 4.5	77.7 ± 9.6	73.3 ± 19.8	74.9 ± 21.3	52.1 ± 20.4	59.3 ± 19.8
Fold-3	Benchmark	649	78.1 ± 22.4	60.9 ± 20.2	80.7 ± 19.8	62.0 ± 19.9	57.9 ± 27.6	57.8 ± 31.8	42.0 ± 24.1	48.9 ± 25.4
	CoSinGAN	4	93.5 ± 4.9	76.4 ± 9.1	94.7 ± 2.1	76.7 ± 7.7	63.5 ± 19.2	62.8 ± 22.7	51.0 ± 22.9	59.5 ± 21.4
	IF-CoSinGAN	4	93.2 ± 7.1	77.7 ± 10.5	94.6 ± 3.7	77.2 ± 9.3	66.7 ± 19.7	66.5 ± 23.2	50.5 ± 21.5	58.8 ± 20.0
	RC-CoSinGAN	4	94.1 ± 7.1	81.0 ± 10.1	95.1 ± 3.6	79.7 ± 8.5	64.8 ± 21.6	67.4 ± 23.0	46.6 ± 24.2	54.2 ± 22.5
Fold-4	Benchmark	873	89.9 ± 12.4	74.0 ± 16.2	92.0 ± 8.7	74.8 ± 16.0	48.6 ± 29.8	52.0 ± 32.1	22.8 ± 22.9	25.6 ± 22.4
	CoSinGAN	4	92.2 ± 6.9	75.5 ± 10.0	93.2 ± 4.8	73.9 ± 10.2	65.4 ± 11.7	62.2 ± 15.8	32.8 ± 24.6	40.4 ± 22.9
	IF-CoSinGAN	4	92.4 ± 6.4	74.6 ± 9.6	93.4 ± 4.0	72.6 ± 9.0	70.1 ± 9.3	68.5 ± 13.5	32.6 ± 23.8	40.9 ± 22.9
	RC-CoSinGAN	4	93.4 ± 5.2	77.3 ± 8.7	93.9 ± 3.7	75.6 ± 9.1	73.3 ± 8.0	73.5 ± 13.6	42.3 ± 26.0	49.1 ± 26.6
Avg	Benchmark	704	87.3 ± 15.7	70.5 ± 18.6	89.4 ± 12.7	71.0 ± 17.6	57.7 ± 26.1	57.2 ± 28.8	40.7 ± 24.1	46.7 ± 24.7
	CoSinGAN	4	93.0 ± 6.0	76.5 ± 11.4	94.1 ± 4.0	75.8 ± 9.1	64.1 ± 18.7	60.5 ± 21.3	42.4 ± 24.4	50.0 ± 23.3
	IF-CoSinGAN	4	93.2 ± 5.9	76.8 ± 10.1	94.3 ± 3.8	75.8 ± 9.3	64.7 ± 18.8	61.4 ± 21.3	37.1 ± 23.4	45.0 ± 22.0
	RC-CoSinGAN	4	**93.9 ± 5.6**	**79.5 ± 9.6**	**94.6 ± 3.8**	**78.1 ± 8.9**	**71.3 ± 19.0**	**72.0 ± 20.9**	**47.4 ± 23.7**	**54.8 ± 23.3**

**Table 6:** Quantitative results (Mean±Standard deviation) of 5-fold cross validation and independent testing based on 3D U-Net. For each fold, average DSC and NSD values are reported. The last row shows the average results of 80 (=5 folds × 16 testing cases per fold) testing cases in the COVID-19-CT-Seg dataset and 250 (=5 folds × 50 testing cases per fold) testing cases in the MosMed dataset. The third column shows the number of real labeled CT slices used for training. ↑ denoting a higher score admits better segmentation performance. The entries in bold highlight the best average results.

Subtask	Methods	Slice Number	Left Lung		Right Lung		Infection (COVID-19-CT-Seg)		Infection (MosMed)	
			DSC ↑	NSD ↑	DSC ↑	NSD ↑	DSC ↑	NSD ↑	DSC ↑	NSD ↑
Fold-0	Benchmark	634	53.8 ± 28.4	39.1 ± 18.3	65.5 ± 19.4	47.4 ± 14.3	65.4 ± 23.9	68.2 ± 23.2	51.0 ± 23.2	60.1 ± 22.4
	CoSinGAN	4	59.9 ± 9.8	40.1 ± 6.6	56.6 ± 14.2	32.6 ± 6.9	51.7 ± 21.1	52.3 ± 21.9	37.7 ± 24.0	45.5 ± 23.8
Fold-1	Benchmark	681	40.3 ± 18.7	27.5 ± 12.0	60.1 ± 11.1	41.7 ± 9.9	64.7 ± 21.8	60.6 ± 25.1	49.8 ± 21.3	55.4 ± 20.1
	CoSinGAN	4	60.7 ± 10.2	36.9 ± 6.7	61.7 ± 8.7	34.2 ± 6.2	67.8 ± 19.5	63.7 ± 22.1	52.8 ± 23.7	59.3 ± 23.4
Fold-2	Benchmark	683	80.3 ± 18.8	66.8 ± 18.8	85.2 ± 12.4	68.6 ± 15.1	60.7 ± 27.6	62.5 ± 28.9	46.8 ± 24.2	50.2 ± 24.2
	CoSinGAN	4	59.9 ± 10.9	40.5 ± 6.4	56.0 ± 14.7	30.8 ± 8.9	64.2 ± 19.4	64.4 ± 20.9	39.7 ± 23.4	47.0 ± 23.0
Fold-3	Benchmark	649	79.7 ± 13.6	65.4 ± 14.4	84.0 ± 9.8	67.7 ± 13.0	62.0 ± 27.9	65.3 ± 28.9	44.8 ± 26.6	52.7 ± 25.4
	CoSinGAN	4	59.5 ± 7.1	41.6 ± 5.1	44.5 ± 15.6	24.4 ± 8.2	59.9 ± 21.1	60.5 ± 24.1	51.0 ± 23.5	57.0 ± 22.4
Fold-4	Benchmark	873	72.4 ± 21.1	58.6 ± 20.8	80.9 ± 13.4	63.4 ± 15.9	51.4 ± 30.2	51.9 ± 31.0	16.7 ± 19.5	19.3 ± 19.0
	CoSinGAN	4	57.4 ± 13.4	37.6 ± 7.3	52.6 ± 16.4	33.3 ± 7.5	64.1 ± 15.4	63.2 ± 17.0	43.4 ± 22.8	49.8 ± 22.0
Avg	Benchmark	704	**65.4 ± 25.6**	**51.6 ± 22.9**	**75.3 ± 16.8**	**57.9 ± 17.5**	60.8 ± 26.3	**61.6 ± 27.5**	41.8 ± 26.4	47.6 ± 26.7
	CoSinGAN	4	59.5 ± 10.5	39.3 ± 6.7	54.3 ± 15.3	31.2 ± 8.4	**61.5 ± 20.2**	60.8 ± 21.8	**44.9 ± 24.2**	**51.7 ± 23.6**

**Table 7:** Quantitative results (Mean ± Standard deviation) of different solutions for learning automated COVID-19 infection segmentation based on 2D U-Net. ↑ denoting a higher score admits better segmentation performance. The entries in bold highlight the best average results.

Methods		Infection (COVID-19-CT-Seg)	
		DSC ↑	NSD ↑
Learning with only non-COVID-19 CT scans	Task2-MSD	7.9 ± 11.5	12.9 ± 15.3
	Task2-StructSeg	0.2 ± 0.8	0.6 ± 1.6
	Task2-NSCLC	1.2 ± 2.9	7.3 ± 9.7
Learning with both COVID-19 and non-COVID-19 CT scans	Task3-MSD	51.2 ± 26.8	52.7 ± 27.4
	Task3-StructSeg	57.4 ± 26.6	57.3 ± 28.4
	Task3-NSCLC	52.5 ± 29.6	52.6 ± 30.3
Learning with only COVID-19 CT scans (or slices)	Benchmark	57.7 ± 26.1	57.2 ± 28.8
	CoSinGAN	64.1 ± 18.7	60.5 ± 21.3
	RC-CoSinGAN	**71.3 ± 19.0**	**72.0 ± 20.9**
